# *In vitro* Characterization of Insulin−Producing β-Cell Spheroids

**DOI:** 10.3389/fcell.2020.623889

**Published:** 2021-01-28

**Authors:** Yonela Ntamo, Ebrahim Samodien, Joleen Burger, Nolan Muller, Christo J. F. Muller, Nireshni Chellan

**Affiliations:** ^1^Biomedical Research and Innovation Platform, South African Medical Research Council, Cape Town, South Africa; ^2^Department of Biochemistry and Microbiology, Faculty of Science and Agriculture, University of Zululand, KwaDlangezwa, South Africa; ^3^Division of Medical Physiology, Faculty of Medicine and Health Sciences, Stellenbosch University, Cape Town, South Africa; ^4^National Health Laboratory Service, Anatomical Pathology, Tygerberg Hospital, Cape Town, South Africa

**Keywords:** 3D culture spheroids, β-cell, transmission electron microscopy, insulin secretion, viability

## Abstract

Over the years, immortalized rodent β-cell lines such as RIN, HIT, MIN, βTC, and INS-1 have been used to investigate pancreatic β-cell physiology using conventional two-dimensional (2D) culture techniques. However, physical and physiological limitations inherent to 2D cell culture necessitates confirmatory follow up studies using sentient animals. Three-dimensional (3D) culture models are gaining popularity for their recapitulation of key features of *in vivo* organ physiology, and thus could pose as potential surrogates for animal experiments. In this study, we aimed to develop and characterize a rat insulinoma INS-1 3D spheroid model to compare with 2D monolayers of the same cell line. Ultrastructural verification was done by transmission electron microscopy and toluidine blue staining, which showed that both 2D monolayers and 3D spheroids contained highly granulated cells with ultrastructural features synonymous with mature pancreatic β-cells, with increased prominence of these features observed in 3D spheroids. Viability, as assessed by cellular ATP quantification, size profiling and glucose utilization, showed that our spheroids remained viable for the experimental period of 30 days, compared to the limiting 5-day passage period of INS-1 monolayers. In fact, increasing ATP content together with spheroid size was observed over time, without adverse changes in glucose utilization. Additionally, β-cell function, assessed by determining insulin and amylin secretion, showed that the 3D spheroids retained glucose sensing and insulin secretory capability, that was more acute when compared to 2D monolayer cultures. Thus, we were able to successfully demonstrate that our *in vitro* INS-1 β-cell 3D spheroid model exhibits *in vivo* tissue-like structural features with extended viability and lifespan. This offers enhanced predictive capacity of the model in the study of metabolic disease, β-cell pathophysiology and the potential treatment thereof.

## Introduction

Cell culture has been widely employed over the years to answer biologically relevant questions in various research fields (Figliolini et al., 2014; Zanoni et al., 2016). It provides an opportunity for better understanding physiological processes prior to verifying the findings in *in vivo* models. Cell culture often involves the development and utilization of *in vitro*, laboratory-based models that recapitulate the pathophysiology of disease and evaluate effective therapeutic modalities (Macdonald, 1990; Skelin et al., 2010; Bonnier et al., 2015; Gaskell et al., 2016). In drug screening, conventionally the standard procedure is initiated in an *in vitro* cell culture system before being verified in animal models subsequent to human testing. Besides being resource intensive and costly often findings fail to be translated when tested in humans (Fogel, 2018; Seyhan, 2019; Van Norman, 2019). Hence, one of the important goals in early drug screening is the development of physiological relevant models that can reduce the number of animals utilized (Hirschhaeuser et al., 2010; LaBarbera et al., 2012). Furthermore, the significant decline in therapeutic inventions is partly associated with the over-reliance on the use of reductionist biological models in preclinical drug screening, for instance, the use of immortalized cell lines cultured in two-dimensional (2D) has been reported (Hirschhaeuser et al., 2010; Weiswald et al., 2015; Horvath et al., 2016).

The development of human diseases is governed by complex mechanisms whose scrutiny has proven to be inherently difficult due to the inability to create normal physical and physiological environments and attain fundamental biological mechanisms using conventional 2D systems. The development of three-dimensional (3D) culturing systems, that provides the physical environment needed for cells to grow, differentiate, and interact naturally with each other have proven to be more physiologically relevant. Three-dimensional culture allows for important cellular processes to develop such as cell-cell communication and organization, differentiation and specialization of gene, and protein expression, relevant to long-term culture for chronic or age-related research (Levenberg et al., 2003; Baharvand et al., 2006; Haycock, 2011; Huh et al., 2011; Sabra and Vermette, 2013; Ravi et al., 2015; Jacobi et al., 2017). Briefly, 3D cultures create a physically improved environment in which immortalized cell lines are permitted to grow in fabricated devices or constructs creating 3D structures. These 3D structures mimic both tissue microarchitecture and function, thereby allowing the recapitulation of the disease pathophysiology by enabling the observation of dynamic cell and signaling environments, thus increasing the preclinical value of 3D models in the field of drug discovery and as predictors of potential therapeutic outcomes (Chang and Hughes-Fulford, 2009; Fey and Wrzesinski, 2012; Gauvin et al., 2012; Fennema et al., 2013; Jacobi et al., 2017).

The etiology of diabetes revolves around insulin-producing pancreatic β-cell dysfunction (Law et al., 2014). To date, diabetes research has utilized rodent immortalized β-cell lines, such as the rat insulinoma cells (RIN), hamster pancreatic β-cells (HIT), transgenic C57BL/6 mouse insulinoma cells (MIN), β-tumor cells (βTC), and rat insulinoma cells (INS-1) (Skelin et al., 2010). These cells produce insulin and smaller amounts of other endocrine hormones including amylin, with some showing better responses to glucose than others (Skelin et al., 2010). These cell lines are primarily used in 2D culture models known to be relatively easy to work with in terms of experimental manipulation and analysis. However, in 2D culture they fail to develop the cellular state of equilibrium characteristic of complex multicellular tissues needed for stable long-term culture (Rupnik, 2009; Wikstrom et al., 2012; Amin et al., 2016). Despite their general use, immortalized β-cell culture stability deteriorates over time, mainly due to phenotypic shifts caused by continuous growth governed only by regular passaging and unstable long-term culture (Skelin et al., 2010). In this context, 3D based models stand to evolve as *in vitro* alternatives that not only mimic *in vivo* microenvironment physically but also enable uninterrupted long-term dynamic cell growth without the need for passaging allowing uninterrupted cell-cell interaction and the development of more tissue-specific morphologies which is more representative of β-cell physiology. In turn, this would greatly aid studies of cell-based diabetes therapies, and other diseases as well, especially those related to chronic long-term treatment. Of all β-cell lines currently available for research, INS-1 cells are considered as one of the most physiologically relevant, with an ability to maintain a differentiated phenotype for up to 6 months in culture (Hohmeier et al., 2000; Skelin et al., 2010; Amin et al., 2016). In this study, we modified a method by Fey and Wrzesinski (2012) to develop an insulin producing 3D INS-1 model. In our model, we evaluated the ultra-structural features, assessed the viability and determined the spheroid functionality.

## Materials and Methods

### Cell Culture

Rat insulinoma-derived insulin−producing INS-1 β-cells used in this study were a kind gift from Professor Luc Bouwens, Vrije Universtiteit, Brussels, Belgium. INS-1 cells were thawed from liquid nitrogen storage and seeded at 8 × 10^5^ cells per 75 cm^2^ culture flask (Sigma-Aldrich, Cat no: CLS3276), containing complete growth medium: RPMI-1640 media (Lonza, Verviers, Belgium; Cat no. BE12-115F) containing L-glutamine and 11 mM glucose, supplemented with 10% fetal bovine serum (FBS) (Gibco, Carlsbad, CA; Cat. no. 31885-023) under standard tissue culture conditions (37°C, 5% CO_2_ in humidified air). Cells were grown for at least three passages before any experiments could be started with media exchanged every 2–3 days. Sub-cultures of INS-1 β-cells were created by scraping the adhered cells in the 75 cm^2^ culture flasks when a confluency of 70–80% was reached and cells were used for a maximum of 20 passages.

#### Two-Dimensional Monolayers and Three-Dimensional Spheroids Preparation

INS-1 cells were seeded at 1 × 10^4^ cells per well into a 96-well plate to form 2D monolayers and incubated for 3–4 days at standard culture conditions. For 3D spheroids, INS-1 cells were seeded at 7.2 × 10^6^ cells per well into AggreWell^TM^ 400Ex plates (Stemcell Technologies, Grenoble, France; Cat. no. 27845) which were centrifuged (100 × g; 3 min) and incubated overnight to form cell aggregates (37°C, 5% CO2 in humidified air). The next day, formed cell aggregates were gently dislodged from the AggreWell^TM^ plate using a disposable Pasteur pipette and collected into a Petri dish containing complete growth media. Compact, intact cell aggregates were hand-picked and transferred into ProtoTissue^TM^ bioreactors (CelVivo ApS, DK-5491 Blommenslyst, Denmark; Cat. no 010) to facilitate the formation of spheroids. The spheroids were incubated in a rotating BioArray Matrix (BAM) system (CelVivo ApS, Denmark) at standard culture conditions for 30 days, with media exchanged every 2–3 days.

#### Transmission Electron Microscopy (TEM)

Scraped INS-1 2D monolayers and collected 3D spheroids were fixed in 2.5% glutaraldehyde in 0.1 M phosphate buffer pH 7.2 at 4°C for 12 h. After fixation the monolayers and spheroids were post-fixed in 3% osmium tetroxide in Palade’s buffer pH 7.4, dehydrated through ascending concentrations of ethanol into Spurr’s resin and the resin allowed to polymerized overnight at 60°C. Semi-thin (1 μM) and Ultra-thin (100 nm) sections of the monolayers and spheroids were cut using Leica EM UC7 ultra-microtome (Vienna, Austria) fitted with a glass knife. Semi-thin sections were stained with 1% toluidine blue and 1% sodium tetraborate solution using a modified method from Burns (1978). The ultra-thin ribbon sections were cut and collected from the water surface onto 200G copper grids and stained with 2% uranyl acetate and Reynolds lead acetate (Sabatini et al., 1963), respectively. The stained ultra-thin sections of both INS-1 cells monolayers and 3D spheroids were imaged using a Joel JEM 1011 (Akishima, Tokyo) electron transmission microscope.

#### Viability Assays

To quantify cellular Adenosine triphosphate (ATP) as a measure of viability in the 2D monolayers, 75 μl of the CellTiter-Glo^®^ reagent was added to each well of a white walled 96-well plate and incubated under standard tissue culture conditions for 30 min and shaken (200 rpm, 37°C) every 10 min to lyse the cells. For 3D spheroids, approximately 10 spheroids per assay were samples and incubated with 100 μl of the CellTiter-Glo^®^ reagent following the manufacturer’s instructions. Luminescence of the cellular ATP in the cell lysates for both 2D monolayers and 3D spheroids was measured using the SpectraMax i3 multimode plate reader (Molecular Devices, California, United States). Cellular ATP was normalized to the protein content of each well, as determined by the Bradford protein quantification assay.

#### Protein Quantification

The Bradford assay (Bio-Rad; Cat no: 500-0203) was used to determine protein content. Briefly, 5 μL sample of cell lysate (section “Viability Assays”) from each well was transferred to a clear, flat-bottom 96-well assay plate. Thereafter, 200 μL Bradford reagent was added to each well followed by 10 min incubation at room temperature. Absorbance was measured at 570 nm on a SpectraMax i3 multimode plate reader. Protein content values were extrapolated from a BSA-generated standard curve.

#### Glucose Stimulated Insulin Secretion Assay

Insulin and amylin secretion in response to glucose stimulation were studied in both 2D monolayers and 3D spheroids. After a 30 min equilibration period with basal glucose media (2.6 mM glucose) at standard culture conditions, INS-1 2D monolayers and 3D spheroids were further incubated with 2.6 mM glucose in media for 90-min. Following this, INS-1 2D monolayers and 3D spheroids were exposed to 16.7 mM glucose (stimulated conditions) for an additional period of 90 min. The media was collected and stored at -80°C.

##### Insulin and Amylin Secretion Assay

Insulin and amylin secretion into the media was determined using a rat/mouse insulin ELISA (Merck Millipore, Cat no: EZRMI-13K) and amylin ELISA Kit (Elabscience, Cat no: E-EL-R2448) according to the manufacturer’s instructions. Levels of secreted insulin and amylin in the INS-1 2D monolayers and 3D spheroids were normalized to cellular protein content, which was determined using a Bradford assay as described above.

#### Glucose Utilization of Spheroids

Glucose concentration in culture media was measured using a One Touch^®^ Select^®^ glucometer and glucose utilization calculated by determining the difference in glucose concentration between the cell culture media before being added to the bioreactor and after removal.

#### Spheroid Size Profiling

Three spheroids were collected on days 1, 4, 10, 19, and 30) into a petri-dish with complete growth media. Spheroids were visualized and captured using a light microscope (Nikon Instruments Inc., Walt Whitman Road Melville; New York). For analysis, the spheroid diameter was measured, and total surface area of each demarcated spheroid was calculated with Image J (Schindelin et al., 2012).

### Statistical Analysis

All experiments were performed in triplicate and the data expressed as mean ± standard error of the mean (SEM). One-way ANOVA was used to compare differences between 2D monolayers and 3D spheroids, with Student’s paired two-tailed *t*-test where applicable. Differences between samples in 3D spheroids were analyzed using the Student’s paired two-tailed *t*-test and One-way ANOVA. *P* < 0.05 was defined as statistically significant.

## Results

### Ultrastructural Differences Between INS-1 Cells Cultured in 2D and 3D

**FIGURE 1:**
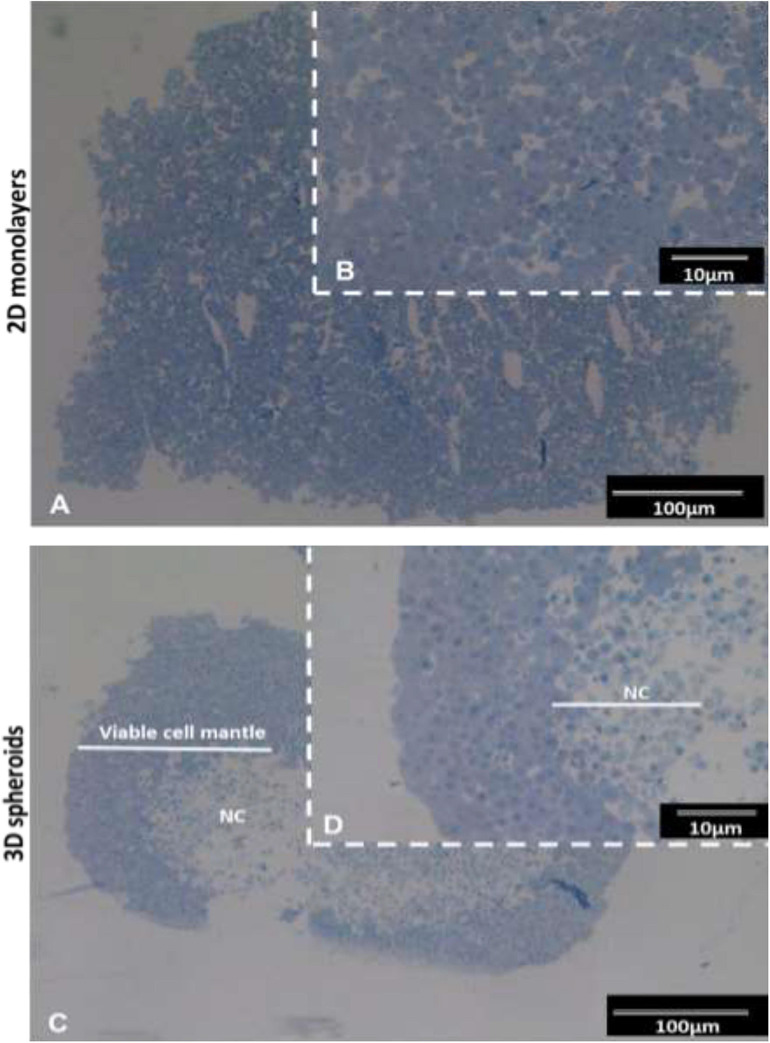
Histological images of INS-1 2D monolayers and 3D spheroid semi-thin sections stained with toluidine blue. Sections of 2D monolayers revealed morphological features such as euchromatic nucleic and prominent nucleoli typical of protein producing cells **(A,B)**. 3D spheroids displayed an outer mantel of viable cells with euchromatic nuclei prominent nucleoli and a central necrotic core (NC) **(C,D)**. Sections captured using 10× and 40× objectives.

The semi-thin resin sections stained with toluidine blue exhibited typical features of viable cells with euchromatic nuclei and prominent nucleoli typical of active protein synthesizing cells (Figure 1). In the 3D spheroids, a clear delineation between the outer viable cell mantel and the apoptotic/necrotic core is observed (Figures 1C,D). The viable cell outer mantel showed similar thickness across the spheroid and between spheroids (Figure 1D).

**FIGURE 2:**
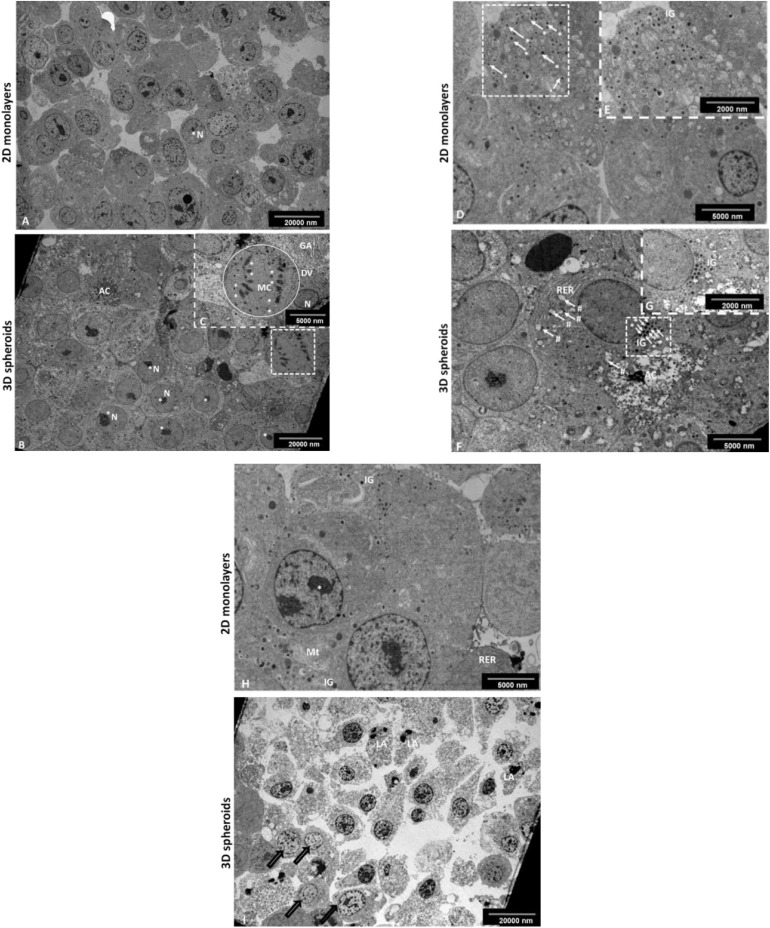
Transmission electron micrograph of the INS-1 2D monolayer and 3D spheroids. Electron micrographs demonstrate some characteristic ultrastructural features synonymous with pancreatic β-cells. In 2D monolayers, cells presented with euchromatic nuclei containing multiple prominent nucleoli **(A)**. In 3D spheroids, cells displayed euchromatic nuclei with prominent nucleoli **(B)**; the occasional mitotic cell with chromosomes evident, RER and Golgi apparatus (insert, **C**). AC, Apoptotic cell; RER, Rough endoplasmic reticulum; N, Nucleus; *Nucleolus, **Chromosomes, Circle DV, dividing cell; GA, Golgi apparatus. Scale bars = 2,000 and 5,000 nm. Electron micrographs confirming the presence of characteristic insulin-like β granules. In 2D monolayers, electron micrographs demonstrate the presence of many insulin-like secretory β-granules (**D** and insert **E**). In 3D spheroids, cells displayed characteristic insulin-like β granules and the presence of an early apoptotic cell located next to the live cells with cytoplasmic RER and early signs of apoptosis including blebbing of mitochondria (**F** and insert **G**). White arrows (#), mitochondrial blebbing; AC, Apoptotic cell; RER, Rough endoplasmic reticulum; white arrows (*) IG, Insulin secretory β granules. Scale bars = 2,000 and 5,000 nm. In 2D monolayers, transmission electron micrograph showed the presence of ultrastructural features including of euchromatic nuclei, rough endoplasmic reticulum, mitochondria, and IG **(H)**. In 3D spheroids, EM showed an area of dead cells in various advanced stages of apoptosis marked with bold arrows **(I)**. Scale bars = 5,000 and 20,000 nm.

Transmission electron microscopy (TEM) was used to evaluate the ultrastructural features of the 2D monolayers and 3D spheroids (Figure 2), respectively. Notably, both the 2D and 3D spheroids expressed ultra-structural features synonymous with pancreatic β-cells *in vivo* including the presence of neuroendocrine granules characteristic of mature β-cells (Figures 2B,C,G,H). These insulin-like secretory β-granules contained a crystalloid electron−dense core surrounded by a membrane separated from the core by a typical halo (Figures 2G,H). These granules were also visible in 2D monolayers (Figures 2B,C). Other structural characteristics typical of protein-producing cells included the presence of rough endoplasmic reticulum (RER), Golgi apparatus, euchromatic nuclei with multiple prominent nucleoli and numerous mitochondria, important for cellular turnover processes throughout β-cell lifespan (Figure 2F). Mitotic cells were occasionally observed, confirming the cell turnover activity within the outer mantel (Figure 2F). Finally, the central core of the 3D spheroids presented with an area of dead cells at different stages of apoptosis and cellular debris (Figure 2I), previously depicted in Figure 1C, while fewer dying cells were observed in 2D monolayers, as expected (Figure 2D).

### Viability Measurement of 2D Monolayer and 3D Spheroids

**FIGURE 3:**
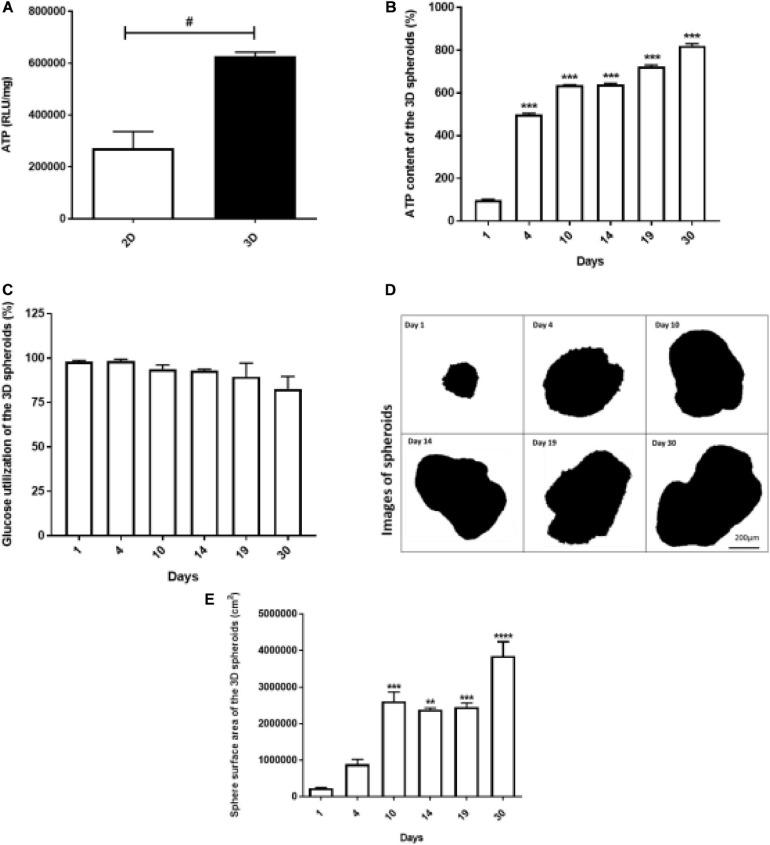
Viability of 2D monolayers and 3D spheroids. The ATP luminescence was assessed in the 2D monolayers and 3D spheroids after 24 h and 1 day in culture, respectively. ATP/mg was higher in the 3D spheroids compared to 2D monolayers **(A)**. In the 3D spheroids, ATP content increased from day 1 to 30 **(B)**, while glucose utilization was comparable throughout **(C)**. The sphere surface area of the spheroids increased from day 1 to 10 and stabilized from day 14 to 19, followed by a gradual increase up to day 30 **(D,E)**. Scale bars: 200 μm **(D)**. Data expressed as mean ± SEM, Student’s *t*-test and ANOVA was used to conduct analysis: ^#^*p* < 0.05 2D vs. 3D condition (Two-way ANOVA); **p < 0.001 vs. day 1, ***p < 0.0001 vs. day 1 (Ordinary One-way ANOVA).

Adenosine triphosphate (ATP) levels were found to be higher in 3D spheroids compared to 2D monolayers (*p* < 0.05) (Figure 3A). The ATP content of the spheroids increased exponentially from day 1 (100% ± 2.35) to day 4 (500.4% ± 4.71), i.e., 1.2-fold increase. From day 5 to 30, a further 1–2-fold daily increase in ATP content was further recorded (Figure 3B). Glucose utilization was unaltered during the period (Figure 3C).

### 3D Spheroid Size Profiling

At various time points (1, 4, 10, 14, 19, and 30 days), spheroids were collected from culture, imaged using a light microscopy to monitor their growth at different days (Figure 3D). Figure 3D shows representative optical images of spheroids and the spheroids surface area is shown in Figure 3E. The areas of the spheroids increased sequentially from day 1 to 10 proportional to the increases in ATP content (Figures 3E,B, respectively). Thereafter, the surface area of the spheroids reached a plateau between day 14 and 19, followed by an increase up to day 30 (Figure 3E).

### INS-1 Spheroid Insulin and Amylin Secretion After Glucose Stimulation

**FIGURE 4:**
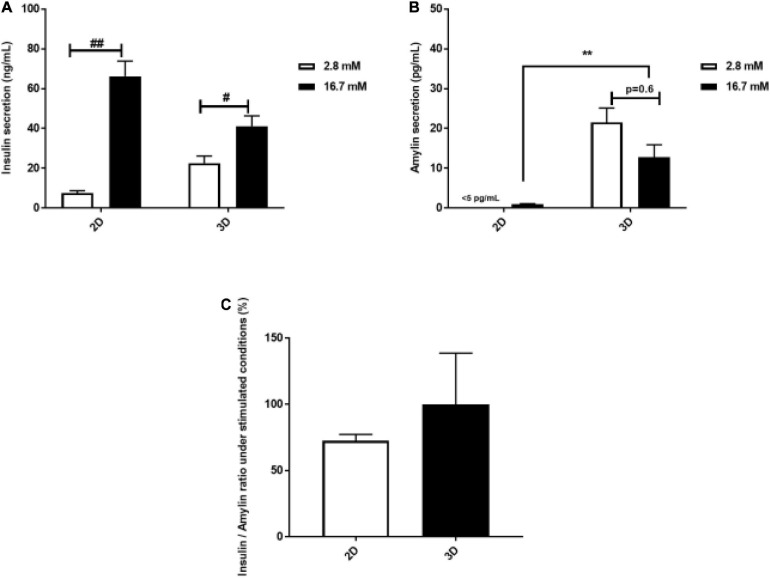
Glucose stimulated insulin and amylin secretion. The amount of insulin secreted by the 2D monolayers, after 4 days culture compared to 3D spheroids after 30 days culture was increased in response to glucose stimulation **(A)**. Amylin secretion by 3D spheroids was slightly increased in response to glucose levels **(B)**. In 3D spheroids, amylin secretion in response to glucose stimulation was higher than in 2D monolayers **(B)**, while insulin to amylin ratio was not statistically significant between 2D monolayers and 3D spheroids **(C)**. Data is represented as mean ± SEM of three independent experiments: ^#^*p* < 0.05; ^##^*p* < 0.01 basal vs. stimulation (Student’s *t*-test); **p < 0.01 2D vs. 3D condition (One-way ANOVA).

The rapid response of β-cells to fluctuating glucose concentrations of is a hallmark of healthy β-cell function. Insulin and concomitant amylin secretion of INS-1 cells cultured in 2D monolayers and 3D spheroids in response to basal (2.6 mM) and stimulated glucose concentrations (16.7 mM), respectively was assessed. Secreted insulin was detected both in 2D monolayers and 3D spheroids (Figure 4A). In 2D monolayers, glucose stimulation resulted in an 8-fold increase in insulin secretion compared to basal glucose (72.19 ng/mL ± 10.69 vs. 8.40 ng/mL ± 1.66, *p* = 0.002) (Figure 4A). In the 3D spheroids, glucose stimulation resulted in a 1.8-fold increase in insulin secretion compared to basal (41.08 ng/mL ± 5.23 vs. 22.6 ng/mL ± 3.49, *p* = 0.03) (Figure 4A). Additionally, insulin secretion in response to glucose stimulation was found to be 1.8-fold lower in 3D spheroids compared to 2D monolayers (Figure 4A). Of note, amylin secretion was not detectable in 2D monolayers (<5 pg/mL) during basal stimulation while 3D spheroids secreted amylin at the same conditions (Figure 4B). In the 3D spheroids, amylin secretion decreased by 1.7-fold at stimulated glucose levels compared to basal (21.5 pg/mL ± 3.57 vs. 12.8 pg/mL ± 3.08, *p* = 0.6) (Figure 4B). A comparison of amylin secretion at stimulated glucose levels showed a 12-fold increase in 3D spheroids compared to 2D monolayers (Figure 4B). While, no statistical significance was observed in the insulin to amylin ratio following glucose stimulation (Figure 4C).

## Discussion

Decreased functional pancreatic β-cell mass is a hallmark for worsening type 2 diabetes and is responsible for relative insulin insufficiency, thus resulting in dysregulated glucose metabolism and hyperglycemia. Pancreatic β-cells play a crucial role in regulating cellular metabolism by secreting insulin in a tightly regulated manner in response to a wide variety of stimuli, with glucose being foremost (Henquin, 2005; Poitout et al., 2010; Marchetti et al., 2017; Moullé et al., 2017). Several *in vitro* pancreatic β-cells cell lines such as RIN, HIT, MIN, βTC, and INS-1 have been used to investigate molecular mechanisms underlying β-cell function and dysfunction (Skelin et al., 2010). These cell lines have been mostly employed as flat cultures (2D conventional culture), which do not entirely mimic human β-cell physiology (Baharvand et al., 2006; Huh et al., 2011). Cell structure, behavior, and function are vital in ensuring that the responses observed *in vitro* closely represents tissue responses *in vivo*. As such interest has increased in the use of 3D cell culture models, with the resultant spheroids having the potential to better predict physiological responses of β-cells and to study long-term repeat dose studies (Baharvand et al., 2006). However, no β-cell spheroid model has been developed that has been structurally and functionally compared with standard 2D cultured INS-1 β-cells. The 3D *in vitro* pancreatic INS-1 β-cell model described in this paper offers a physiologically relevant microenvironment for islet-like cluster formation and maintenance of β-cell function. We first modified a technique previously used for creating C3A liver spheroids (Fey and Wrzesinski, 2012) and further characterized the morphological and ultra-structural features of the INS-1 3D spheroids generated. On an ultrastructural level, the spheroids retained the ability to produce membrane-bound insulin β-granules as seen in the EM micrographs; the granules were also seen in the 2D monolayers (Figures 2G,H). Thus the spheroids can still be identified as typical β-cells due to the presence of distinct β-granules (Sato et al., 1966; Orci, 1985; Wrenshall, 2006; Ivanova et al., 2013). In the 2D monolayers (Figures 2C,D), some β-granules appeared to be less electron dense and thus immature, supporting previous reports (Hutton, 1989, 1994; Masini et al., 2012; Stephens et al., 2017; Ferri et al., 2019). In the 3D spheroids, these β-granules presented with an electron dense core associated with the co-storage of insulin and zinc in a crystallized form (Figures 2G,H), which was associated with their maturity compared to the 2D monolayers. Indeed, studies have described that during active insulin biosynthesis the electron dense crystalline core forms as insulin aggregates with Zn^2+^ ions form Zn-hexameric complexes (Suckale and Solimena, 2010; Li, 2014). The quantity of insulin granules observed in 2D monolayers correlates with increased insulin secretion observed during glucose stimulation when compared to the 3D spheroids (Figure 4A). Although, differential responses to glucose stimulation existed between 2D monolayers and 3D spheroids, cells cultured in both systems generally retained the ability of β-cells *in vivo* to respond to glucose stimulation with insulin release (Figure 4A). The ratio of stimulated insulin secretion in the 3D culture system compared to basal was more comparable to that described *in vivo* in humans where healthy individuals have been described to secrete 18 and 40 U/day with half of this amount secreted in the basal state (Ramchandani et al., 2010). These data are consistent with findings reported by many on stimulation of insulin secretion in response to glucose using *in vivo* models and human studies (Zhang et al., 2004; Moynihan et al., 2005; Pi et al., 2007; Saadeh et al., 2012).

Observations of RER, Golgi apparatus, euchromatic nuclei, and prominent nucleoli in both 2D monolayers and 3D spheroids in our study (Figures 2A,E), were indicative of active cellular protein synthesis and is consistent with previous findings (Sato et al., 1966; Lowe and Anderson, 2015). The observation of a well-developed RER was closely associated with insulin release data (Figure 4) as studies have shown that RER is vital for insulin production, modification and secretion via the trans Golgi network (i.e., Golgi apparatus and secretory granules). The fact that both our 2D monolayers and spheroids displayed euchromatic nuclei, multiple nucleoli and mitochondria was indicative of normal cell function, including the integration and generation of metabolic signals for glucose homeostasis (Brun and Maechler, 2016; Jin and Diano, 2018; Schöfer and Weipoltshammer, 2018). Data from our toluidine blue stained and TEM semi-thin sections of 3D spheroids confirmed various cell cycling phases (Figures 1C,D, 2I). These findings are consistent with literature that in 3D culture, unlike 2D culture, cells are usually found in various cell cycling stages (such as proliferation, quiescence, apoptosis, hypoxia, and necrosis) as is seen *in vivo* (Khaitan et al., 2006; Hirschhaeuser et al., 2010; Mehta et al., 2012). The presence of occasional apoptotic and mitotic cells, close to the periphery of the 3D spheroids was suggestive of active cellular turnover, further supporting the notion above (Figure 2F). The central core of the spheroids comprised an area of apoptotic cells and cellular debris (Figures 1C, 2I), consistent with ischemic stress and/or nutrient deficiency. The viable cell mantel depth of ca. 100 μm is as expected consistent with the O_2_ diffusion distance in tissue (Place et al., 2017). Our 2D monolayers toluidine blue data showed a majority of viable cells only (Figures 1A, 2A), consistent with growth dynamics of passaged cells which remain in a proliferative state (Khaitan et al., 2006; Mehta et al., 2012). These ultra-structural features demonstrate how 2D flat culture often fails to recapitulate *in vivo* cellular microarchitecture, with the spheroids providing an intermediate closer to what is seen *in vivo*.

Our cell viability data showed that 3D spheroids remained viable for up to 30 days in culture, unlike the 2D cultured INS-1 monolayers which required sub-culturing after 72–96 h. The process of subculturing has been shown to disrupt normal cell function and in fact, can damage cell-cell contact/communication (Jung et al., 2009; Batista et al., 2010; Wrzesinski and Fey, 2013). Sustained viability over the extended 30-day period was concomitant to sustained ATP synthesis in the spheroids (Figure 3B). This indeed, highlights one of the many advantages of 3D culture which is the ability to grow cells for an extended period of time without chemical or physical detachment, thus potentiating a model for long-term studies *in vitro*. A data comparison of the ATP content normalized to protein content between the 2D monolayers and 3D spheroids (Figure 3A), showed that cells in the 3D spheroids had increased cellular energy levels compared to those in 2D monolayers, which is indicative of increased cellular metabolism and is imperative for insulin synthesis (Levenberg et al., 2003; Haycock, 2011; Ravi et al., 2015; Jacobi et al., 2017). Theoretically, an increase in cellular ATP is expected to require increased glucose usage accompanied by anaerobic glycolysis, however, this was not the case in our study, contrasting with previous reports (Khaitan et al., 2006) which may be as a result of discrepancies between glucose removed from the media and that which was actually internalized by the cells. In our study, the ATP assay serves as a crude measure of cellular metabolism and viability. In future work, we plan on expanding the investigation into these cellular processes by exploring the roles of oxidative stress and anaerobic respiration in glucose utilization. Furthermore, intracellular glucose taken up should be determined in order to fully represent the gluco-metabolic profile of the cells.

The increases in spheroid surface area (Figures 3E,F) provided a confirmation of the ability of the spheroids to adequately respond to glucose, produce ATP and promote cell growth and replication. The observation that on days 14–19 the spheroid growth measured in terms of the sphere surface area appeared to stabilized or slightly decrease but without impacting on ATP content (Figures 3F,B, respectively). This implies that the spheroids were still metabolically active (Khaitan et al., 2006; Bussador do Amaral et al., 2010) and that perhaps the size decrease was as a result of contraction of the interconnected cell network, as opposed to overt cell death. We postulated that, the sphere surface area reductions could be due to late apoptotic cellular shrinkage (pyknosis), karyorrhexes within the core and cytoskeleton intercellular proteins contraction observed by the presence of more cell-cell junctions within the viable mantle similar to explanations by Papadopoulos et al. (2000); Cheng et al. (2009), Sodek et al. (2009) and Huang et al. (2013).

To further compare and contrast the functionality of cells grown as 2D monolayers and 3D spheroids, we quantified the secretion of amylin. In essence, amylin and insulin are secreted in response to glucose, where amylin secretion causes a fall in glucagon levels which in turn maintains normoglycemia during the postprandial period (Dégano et al., 1993; Lutz, 2012; Zhang et al., 2016; Chwalba et al., 2019). Our data has shown a differing secretion pattern between amylin and its co-stored/secreted partner insulin, in response to glucose stimulation. The 3D spheroids were able to secrete detectable levels of amylin at basal glucose levels, unlike 2D monolayers in our study and thus boasts another advantage over conventional 2D monolayers of β-cells (Figure 4B; Diti, 2018). A rare, unlikely observation was that although our 3D spheroids secreted amylin, they failed to increase their secretion following a glucose stimulation (Figure 4B). This is in contrast with literature that shows that amylin secretion correlates with insulin secretion at a ratio of 1:100 (Moore and Cooper, 1991; Zhang et al., 2016). Other studies have, however, described a lack of co-secretion between insulin and amylin from isolated pancreatic islets *in vitro* (Dégano et al., 1993; Stridsberg et al., 1993) suggesting that our spheroids behave more like isolated islets than 2D cultured β-cells. It is, however, important to highlight that, in this regard our model will not aim at replacing islet transplantation therapy but will provide invaluable knowledge for functional studies where mimicking the structure and composition of natural tissues in order to achieve a similar functional outcome is of particular interest. Several challenges in studies utilizing isolated islets have been reported by many researchers, and these include ethical concerns since humans and rodents are involved, the donor shortages, the intricacy of the isolation process and the stability of isolated islets in culture (Hart and Powers, 2019; Nano et al., 2019). In this regard, our 3D spheroid model promises to be a more practical alternative as these can be largely made available with ease and enhanced viability in culture and offers a room for improvements to enhance applicability of research objective. Considering the result that, 2D monolayers were highly responsive in terms of insulin secretion, the small amounts of amylin secreted by the monolayers was a surprising result (Figure 4). Indeed, differential response to insulin secretion agonists in β-cells cultured under 2D and 3D systems have been reported and may explain the difference we observed in amylin as the two hormones are co-secreted (Figure 4B; Ramachandran et al., 2015; Amin et al., 2016). In addition, due to their non-clonal nature, discrepancies in variable responses to glucose-stimulated insulin secretion in INS-1 cells have been described (Merglen et al., 2004). An important contributor that is required for proper β-cell function is cell-to-cell communication (Hauge-Evans et al., 1999; Hohmeier et al., 2000; Merglen et al., 2004). We hypothesize that the strong dynamic secretory responses observed from INS-1 cells grown as 3D spheroids may have been due to the presence of cell-cell communication, however, this will need to be assessed in future studies. In essence, this indicates that 3D spheroids displayed cell-to-cell communication properties required for proper function. These 3D spheroids did, however, also display notable differences in comparison with native β-cells in terms of amylin secretion which warrants further investigation.

Our data, presented in this study, collectively compared ultrastructural components and cellular responses of INS-1 pancreatic β-cells cultured as 2D monolayers or as 3D spheroids. A range of assays were used to characterize the dynamic responses, where we showed that INS-1 cells cultured as 3D spheroids more closely mimicked β-cells *in vivo* compared to 2D monolayers as evident in improved biochemical processes, prolonged viability in culture and cellular heterogeneity. Using this 3D culturing method could ultimately hold great benefits in disease research related to chronic/long-term effects or treatments and could alleviate reliance on animal models for these purposes.

## Conclusion

In the present study, we successfully developed a reproducible technique for creating 3D INS-1 pancreatic β-cell spheroids. Our data revealed that the 3D spheroids displayed *in vivo*-like ultra-structural features of β-cells with the ability to secrete insulin and amylin in response to glucose stimulation. This 3D cultured INS-1 spheroid model not only represents significant improvement on conventional 2D monolayers but may also represent an alternative intermediate to the use of sentient animals in research. Our spheroid model holds promise as a novel *in vitro* β-cell model for studying gluco-metabolic related diseases over longer periods of time.

## Data Availability Statement

All datasets generated for this study are included in the article/supplementary material, further inquiries can be directed to the corresponding author/s.

## Author Contributions

YN: writing—original draft, investigation, methodology of 3D related experiments, and writing—review and editing. JB: methodology 2D related experiments and writing—review and editing. ES: writing—review and editing and visualization. CM: visualization, supervision, and writing—review and editing. NC: supervision, conceptualization, visualization, writing—review and editing, and funding acquisition. All authors contributed to the article and approved the submitted version.

## Conflict of Interest

The authors declare that the research was conducted in the absence of any commercial or financial relationships that could be construed as a potential conflict of interest.
