# Corrigendum: *In vitro* Characterization of Insulin–Producing β-Cell Spheroids

**DOI:** 10.3389/fcell.2021.662574

**Published:** 2021-03-03

**Authors:** Yonela Ntamo, Ebrahim Samodien, Joleen Burger, Nolan Muller, Christo J. F. Muller, Nireshni Chellan

**Affiliations:** ^1^Biomedical Research and Innovation Platform, South African Medical Research Council, Cape Town, South Africa; ^2^Department of Biochemistry and Microbiology, Faculty of Science and Agriculture, University of Zululand, KwaDlangezwa, South Africa; ^3^Division of Medical Physiology, Faculty of Medicine and Health Sciences, Stellenbosch University, Cape Town, South Africa; ^4^National Health Laboratory Service, Anatomical Pathology, Tygerberg Hospital, Cape Town, South Africa

**Keywords:** 3D culture spheroids, β-cell, transmission electron microscopy, insulin secretion, viability

In the original article, there was an error. The name of the suppliers of the ProtoTissue^TM^ bioreactors and BioArray Matrix (BAM) system were incorrectly provided as the suppliers have pointed out the error to the authors.

A correction has been made to *Materials and method section; subsection 2.1.1*. *Two-dimensional* *monolayers and three-dimensional spheroids*.

Compact, intact cell aggregates were hand-picked and transferred into ProtoTissue™ bioreactors (CelVivo ApS, DK-5491 Blommenslyst, Denmark; Cat. no 010) to facilitate the formation of spheroids. The spheroids were incubated in a rotating BioArray Matrix (BAM) system (CelVivo ApS, Denmark) at standard culture conditions for 30 days, with media exchanged every 2–3 days.

The authors apologize for this error and state that this does not change the scientific conclusions of the article in any way. The original article has been updated.

